# Trimesic acid dimethyl sulfoxide solvate: space group revision

**DOI:** 10.1107/S1600536808018655

**Published:** 2008-06-28

**Authors:** Sylvain Bernès, Guadalupe Hernández, Roberto Portillo, René Gutiérrez

**Affiliations:** a DEP Facultad de Ciencias Químicas, UANL, Guerrero y Progreso S/N, Col. Treviño, 64570 Monterrey, NL, Mexico; b Laboratorio de Síntesis de Complejos, Facultad de Ciencias Químicas, Universidad Autónoma de Puebla, AP 1067, 72001 Puebla, Pue., Mexico

## Abstract

The structure of the title solvate, C_9_H_6_O_6_·C_2_H_6_OS, was determined 30 years ago [Herbstein, Kapon & Wasserman (1978 ▶). *Acta Cryst*. B**34**, 1613–1617], with data collected at room temperature, and refined in the space group *P*2_1_. The present redetermination, based on high-resolution diffraction data, shows that the actual space group is more likely to be *P*2_1_/*m*. The crystal structure contains layers of trimesic acid molecules lying on mirror planes. A mirror plane also passes through the S and O atoms of the solvent molecule. The molecules in each layer are inter­connected through strong O—H⋯O hydrogen bonds, forming a two-dimensional supra­molecular network within each layer. The donor groups are the hydroxyls of the trimesic acid mol­ecules, while the acceptors are the carbonyl or the sulfoxide O atoms.

## Related literature

For the first report on the title solvate structure, see: Herbstein *et al.* (1978 ▶). For the use of trimesic acid as a building block for supra­molecular networks, see: Almeida Paz & Klinowski (2004 ▶). For a description of hydrogen bonds, see: Desiraju & Steiner (1999 ▶).
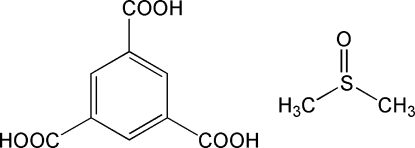


## Experimental

### 

#### Crystal data

C_9_H_6_O_6_·C_2_H_6_OS
*M* *_r_* = 288.27
Monoclinic, 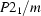

*a* = 8.7444 (7) Å
*b* = 6.8365 (7) Å
*c* = 10.7113 (8) Å
β = 96.195 (5)°
*V* = 636.59 (10) Å^3^
*Z* = 2
Mo *K*α radiation
μ = 0.28 mm^−1^
*T* = 298 (1) K
0.60 × 0.48 × 0.36 mm

#### Data collection

Siemens P4 diffractometer
Absorption correction: ψ scan (*XSCANS*; Siemens, 1996 ▶) *T*_min_ = 0.851, *T*_max_ = 0.904
4582 measured reflections
2007 independent reflections
1772 reflections with *I* > 2σ(*I*)
*R*_int_ = 0.015
3 standard reflections every 97 reflections intensity decay: <1%

#### Refinement

*R*[*F* ^2^ > 2σ(*F* ^2^)] = 0.037
*wR*(*F* ^2^) = 0.112
*S* = 1.07
2007 reflections
123 parameters
H atoms treated by a mixture of independent and constrained refinement
Δρ_max_ = 0.38 e Å^−3^
Δρ_min_ = −0.30 e Å^−3^

### 

Data collection: *XSCANS* (Siemens, 1996 ▶); cell refinement: *XSCANS*; data reduction: *XSCANS*; program(s) used to solve structure: *SHELXS97* (Sheldrick, 2008 ▶); program(s) used to refine structure: *SHELXL97* (Sheldrick, 2008 ▶); molecular graphics: *Mercury* (Macrae *et al.*, 2006 ▶); software used to prepare material for publication: *SHELXL97*.

## Supplementary Material

Crystal structure: contains datablocks I, global. DOI: 10.1107/S1600536808018655/fb2098sup1.cif

Structure factors: contains datablocks I. DOI: 10.1107/S1600536808018655/fb2098Isup2.hkl

Additional supplementary materials:  crystallographic information; 3D view; checkCIF report

## Figures and Tables

**Table 1:** Hydrogen-bond geometry (Å, °)

*D*—H⋯*A*	*D*—H	H⋯*A*	*D*⋯*A*	*D*—H⋯*A*
O1—H1⋯O6^i^	0.84 (3)	1.82 (3)	2.6435 (16)	165 (3)
O3—H3⋯O7^ii^	0.85 (4)	1.83 (4)	2.6593 (17)	164 (4)
O5—H5⋯O7^iii^	0.86 (3)	1.73 (3)	2.5723 (16)	169 (3)

Symmetry codes: (i) 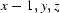; (ii) 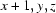; (iii) 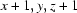.

**Fig. 1.**
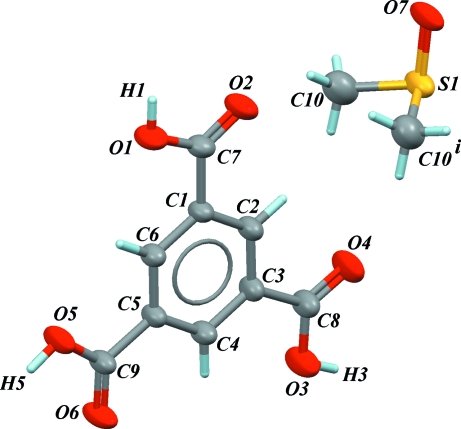
The structure of the title compound, with displacement ellipsoids for non-H atoms at the 50% probability level. Symmetry code: (i) x, 1/2 - y, z.

**Fig. 2.**
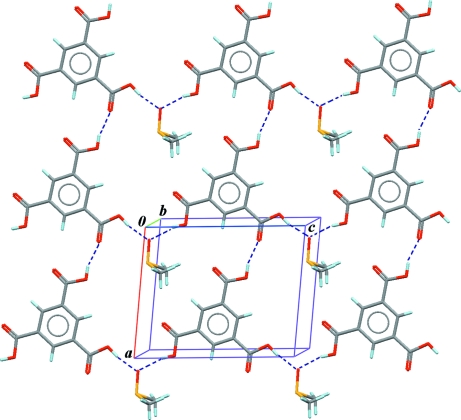
A part of the packing structure for the title compound, viewed approximately along [010]. The hydrogen bonds forming the two-dimensional supramolecular network are depicted by dashed lines.

## supplementary crystallographic information

### Comment

The title compound was obtained during attempts to prepare coordination compounds with transition metals and benzene-1,3,5-tricarboxylic acid. The latter compound is also known as trimesic acid, TMA. It is a rigid, planar molecule that is soluble in a number of solvents. Its three *exo*-carboxylic acid groups are arranged symmetrically around the benzene ring, forming a flat, trigonal molecule, which can be used as a building block in the construction of organic crystals and multidimensional metalorganic frameworks (*e.g*. Almeida Paz & Klinowski, 2004).

The clathration ability of TMA allowed to prepare a number of solvate structures, including hydrates, and consequently determination of these structures. Among them, the dimethylsulfoxide (DMSO) solvate has been reported already 30 years ago (Herbstein *et al.*, 1978). The data were collected at room temperature with Mo-*K*α radiation. Laue symmetry as well as systematic extinctions are in agreement with the space group *P*2_1_/*m* or *P*2_1_. Herbstein *et al.* applied the Hamilton test, *i.e.* essentially based their choice on final *R* residuals. The space group *P*2_1_ was eventually retained (*R* = 0.084) and *P*2_1_/*m* rejected (*R* = 0.092), despite the *E* statistics, which favoured a centrosymmetric space group. The authors, however, commented in their publication that "there is some doubt about the correctness of this decision".

We have now collected an accurate high-resolution diffraction pattern for this compound. Wilson statistics are not in agreement with the non-centrosymmetric space group, for instance 〈*E*^2^-1〉 = 1.002 for 4589 *E *values. Refinement in space group *P*2_1_ converges to *R*_1_ = 0.035 for 1772 *F*_o_>4σ(*F*_o_). However, abnormally high correlation matrix elements are observed for methyl groups in DMSO, and methyl H atoms, if refined freely, exhibit unrealistic C—H bond lengths, ranging from 0.68 Å to 1.48 Å. Finally, refinement using non-merged data (286 measured Friedel pairs) gives an inconsistent Flack parameter, 0.23 (13).

All these symptoms indicate that the space group should be rather *P*2_1_/*m*. All the atoms with exception of the methyl group (C10) of the DMSO molecule lie in the mirror plane. The methyl group (C10) occupies a general position (Fig. 1). Expected geometry for both moieties is observed.

The displacement parameters deserve a careful examination. The longest axes of the displacement parameters are perpendicular to the molecular planes. In the case of the TMA molecule, the *U*_3_/*U*_1_ ratios of non-hydrogen atoms lie in the range 2.17–6.60. A similar thermal behaviour is observed for DMSO atoms lying in the *m* plane, S1 (*U*_3_/*U*_1_ = 3.69) and O7 (*U*_3_/*U*_1_ = 5.77). Such motions suggest another possibility that the crystal can contain statistically distributed non-centrosymmetric domains; *i.e.* the structure can be non-centrosymmetric on a shorter scale. Therefore, the space group *P*2_1_ can not be totally ruled out, and the actual space group may also be dependent on the choice of the particular sample. Further work, like multi-temperatures data collections, would be desirable in order to determine the symmetry unambiguously.

On the other hand, the molecular motion within the molecular planes would be affected by stronger intermolecular interactions that take place within each molecular layer. The molecules are involved in a two-dimensional supramolecular network through the strong hydrogen bonds (Desiraju & Steiner, 1999; Tab. 1). All the hydroxyl groups of the TMA molecule form O—H···O hydrogen bonds using carbonyl and sulfoxide O atoms as acceptors. As a result, the molecular layers are formed in the crystal structure (Fig. 2), parallel to (010). These layers correspond to the crystallographic *m* planes, and are thus separated by *b*/2 = 3.42 Å.

### Experimental

Copper (0.1 g, 1.5 mmol), TMA (0.32 g, 1.5 mmol), and DMSO (3.3 g, 42.2 mmol) were placed in a flask and the mixture was heated at 338 K with magnetic stirring until total dissolution of the metal was observed (0.5–2 hours). The solution was filtered and allowed to stand at room temperature for 12 hours, after which the crystals of the title compound were formed.

### Refinement

Hydroxyl H atoms were found in a difference map, and refined freely. Other H atoms were placed in idealized positions, with C—H bond lengths fixed to 0.93 (aromatic CH) or 0.96 Å (methyl CH_3_) and refined using a riding model approximation, with *U*_iso_(H) = 1.5*U*_eq_(carrier C) for the methyl group and *U*_iso_(H) = 1.2*U*_eq_(carrier C) for the aryl groups. The methyl group is considered as a rigid group free to rotate about the S1—C10 bond.

### Crystal data

**Table:**

C_9_H_6_O_6_·C_2_H_6_OS	*F*_000_ = 300
*M**_r_* = 288.27	*D*_x_ = 1.504 Mg m^−^^3^
Monoclinic, *P*2_1_/*m*	Mo *K*α radiation λ = 0.71073 Å
Hall symbol: -P 2yb	Cell parameters from 45 reflections
*a* = 8.7444 (7) Å	θ = 4.7–13.8º
*b* = 6.8365 (7) Å	µ = 0.28 mm^−^^1^
*c* = 10.7113 (8) Å	*T* = 298 (1) K
β = 96.195 (5)º	Cell measurement pressure: 101(2) kPa
*V* = 636.59 (10) Å^3^	Prism, colourless
*Z* = 2	0.60 × 0.48 × 0.36 mm

### Data collection

**Table:**

Siemens P4 diffractometer	1772 reflections with *I* > 2σ(*I*)
Radiation source: fine-focus sealed tube	*R*_int_ = 0.015
Monochromator: graphite	θ_max_ = 30.0º
*T* = 298(1) K	θ_min_ = 1.9º
*P* = 101(2) kPa	*h* = −12→12
2θ/ω scans	*k* = −9→1
Absorption correction: ψ scan(XSCANS; Siemens, 1996)	*l* = −15→15
*T*_min_ = 0.851, *T*_max_ = 0.904	3 standard reflections
4582 measured reflections	every 97 reflections
2007 independent reflections	intensity decay: <1%

### Refinement

**Table:**

Refinement on *F*^2^	Secondary atom site location: difference Fourier map
Least-squares matrix: full	Hydrogen site location: difference Fourier map
*R*[*F*^2^ > 2σ(*F*^2^)] = 0.037	H atoms treated by a mixture of independent and constrained refinement
*wR*(*F*^2^) = 0.112	*w* = 1/[σ^2^(*F*_o_^2^) + (0.0632*P*)^2^ + 0.1017*P*] where *P* = (*F*_o_^2^ + 2*F*_c_^2^)/3
*S* = 1.07	(Δ/σ)_max_ < 0.001
2007 reflections	Δρ_max_ = 0.38 e Å^−^^3^
123 parameters	Δρ_min_ = −0.30 e Å^−^^3^
20 constraints	Extinction correction: SHELXL97 (Sheldrick, 2008), Fc^*^=kFc[1+0.001xFc^2^λ^3^/sin(2θ)]^-1/4^
Primary atom site location: structure-invariant direct methods	Extinction coefficient: 0.040 (9)

### Fractional atomic coordinates and isotropic or equivalent isotropic displacement parameters (Å^2^)

**Table:**

	*x*	*y*	*z*	*U*_iso_*/*U*_eq_	
C1	0.60335 (14)	0.2500	0.50040 (13)	0.0315 (3)	
C2	0.65053 (16)	0.2500	0.38033 (13)	0.0356 (3)	
H2A	0.5773	0.2500	0.3106	0.043*	
C3	0.80576 (16)	0.2500	0.36384 (12)	0.0355 (3)	
C4	0.91692 (16)	0.2500	0.46759 (13)	0.0361 (3)	
H4A	1.0210	0.2500	0.4567	0.043*	
C5	0.86956 (15)	0.2500	0.58847 (12)	0.0333 (3)	
C6	0.71358 (15)	0.2500	0.60405 (13)	0.0325 (3)	
H6A	0.6829	0.2500	0.6845	0.039*	
C7	0.43539 (16)	0.2500	0.51484 (15)	0.0363 (3)	
C8	0.84857 (18)	0.2500	0.23234 (14)	0.0428 (4)	
C9	0.98606 (16)	0.2500	0.70011 (13)	0.0382 (4)	
O1	0.40892 (13)	0.2500	0.63458 (12)	0.0512 (4)	
H1	0.314 (4)	0.2500	0.640 (3)	0.079 (9)*	
O2	0.33561 (13)	0.2500	0.42797 (12)	0.0527 (4)	
O3	0.99843 (14)	0.2500	0.22789 (12)	0.0648 (5)	
H3	1.018 (4)	0.2500	0.152 (4)	0.105 (12)*	
O4	0.75495 (16)	0.2500	0.14056 (11)	0.0610 (4)	
O5	0.92574 (13)	0.2500	0.80719 (10)	0.0548 (4)	
H5	0.998 (3)	0.2500	0.868 (3)	0.063 (7)*	
O6	1.12357 (13)	0.2500	0.69470 (12)	0.0600 (4)	
S1	0.28919 (4)	0.2500	0.01135 (3)	0.04770 (18)	
C10	0.35278 (17)	0.0539 (3)	0.11302 (15)	0.0600 (4)	
H10A	0.4632	0.0522	0.1249	0.090*	
H10B	0.3158	−0.0677	0.0764	0.090*	
H10C	0.3137	0.0713	0.1927	0.090*	
O7	0.11484 (13)	0.2500	0.00856 (11)	0.0620 (5)	

### Atomic displacement parameters (Å^2^)

**Table:**

	*U*^11^	*U*^22^	*U*^33^	*U*^12^	*U*^13^	*U*^23^
C1	0.0208 (5)	0.0452 (7)	0.0285 (6)	0.000	0.0028 (4)	0.000
C2	0.0258 (6)	0.0547 (9)	0.0257 (6)	0.000	0.0002 (5)	0.000
C3	0.0276 (6)	0.0578 (9)	0.0212 (6)	0.000	0.0037 (4)	0.000
C4	0.0239 (6)	0.0595 (9)	0.0251 (6)	0.000	0.0043 (5)	0.000
C5	0.0229 (5)	0.0545 (8)	0.0226 (5)	0.000	0.0023 (4)	0.000
C6	0.0236 (6)	0.0498 (8)	0.0246 (6)	0.000	0.0045 (4)	0.000
C7	0.0227 (6)	0.0491 (8)	0.0371 (7)	0.000	0.0032 (5)	0.000
C8	0.0327 (7)	0.0727 (11)	0.0236 (6)	0.000	0.0052 (5)	0.000
C9	0.0233 (6)	0.0678 (10)	0.0234 (6)	0.000	0.0025 (4)	0.000
O1	0.0227 (5)	0.0914 (10)	0.0407 (6)	0.000	0.0092 (4)	0.000
O2	0.0261 (5)	0.0874 (10)	0.0433 (7)	0.000	−0.0024 (4)	0.000
O3	0.0320 (6)	0.1391 (16)	0.0244 (5)	0.000	0.0083 (4)	0.000
O4	0.0401 (6)	0.1171 (13)	0.0247 (5)	0.000	−0.0012 (4)	0.000
O5	0.0266 (5)	0.1159 (12)	0.0218 (5)	0.000	0.0026 (4)	0.000
O6	0.0216 (5)	0.1258 (13)	0.0330 (6)	0.000	0.0042 (4)	0.000
S1	0.0287 (2)	0.0895 (4)	0.0254 (2)	0.000	0.00491 (13)	0.000
C10	0.0550 (8)	0.0642 (9)	0.0587 (8)	−0.0010 (7)	−0.0031 (6)	−0.0050 (7)
O7	0.0275 (5)	0.1342 (15)	0.0238 (5)	0.000	0.0010 (4)	0.000

### Geometric parameters (Å, °)

**Table:**

C1—C6	1.3895 (18)	C8—O4	1.2095 (19)
C1—C2	1.3925 (19)	C8—O3	1.3165 (19)
C1—C7	1.4933 (18)	C9—O6	1.2101 (17)
C2—C3	1.3876 (19)	C9—O5	1.3131 (17)
C2—H2A	0.9300	O1—H1	0.84 (3)
C3—C4	1.3952 (19)	O3—H3	0.85 (4)
C3—C8	1.496 (2)	O5—H5	0.86 (3)
C4—C5	1.4014 (18)	S1—O7	1.5217 (12)
C4—H4A	0.9300	S1—C10^i^	1.7781 (17)
C5—C6	1.3918 (18)	S1—C10	1.7781 (17)
C5—C9	1.4849 (19)	C10—H10A	0.9600
C6—H6A	0.9300	C10—H10B	0.9600
C7—O2	1.2049 (19)	C10—H10C	0.9600
C7—O1	1.3277 (19)		
			
C6—C1—C2	119.26 (12)	O1—C7—C1	112.08 (12)
C6—C1—C7	121.50 (12)	O4—C8—O3	124.03 (14)
C2—C1—C7	119.24 (12)	O4—C8—C3	123.31 (15)
C3—C2—C1	120.60 (12)	O3—C8—C3	112.66 (13)
C3—C2—H2A	119.7	O6—C9—O5	122.46 (13)
C1—C2—H2A	119.7	O6—C9—C5	124.08 (13)
C2—C3—C4	120.37 (12)	O5—C9—C5	113.46 (12)
C2—C3—C8	117.87 (12)	C7—O1—H1	110 (2)
C4—C3—C8	121.76 (13)	C8—O3—H3	110 (2)
C3—C4—C5	119.08 (12)	C9—O5—H5	109.3 (19)
C3—C4—H4A	120.5	O7—S1—C10^i^	104.98 (6)
C5—C4—H4A	120.5	O7—S1—C10	104.98 (6)
C6—C5—C4	120.13 (12)	C10^i^—S1—C10	97.87 (11)
C6—C5—C9	119.97 (12)	S1—C10—H10A	109.5
C4—C5—C9	119.91 (12)	S1—C10—H10B	109.5
C1—C6—C5	120.56 (12)	H10A—C10—H10B	109.5
C1—C6—H6A	119.7	S1—C10—H10C	109.5
C5—C6—H6A	119.7	H10A—C10—H10C	109.5
O2—C7—O1	123.97 (14)	H10B—C10—H10C	109.5
O2—C7—C1	123.94 (14)		
			
C6—C1—C2—C3	0.0	C6—C1—C7—O2	180.0
C7—C1—C2—C3	180.0	C2—C1—C7—O2	0.0
C1—C2—C3—C4	0.0	C6—C1—C7—O1	0.0
C1—C2—C3—C8	180.0	C2—C1—C7—O1	180.0
C2—C3—C4—C5	0.0	C2—C3—C8—O4	0.0
C8—C3—C4—C5	180.0	C4—C3—C8—O4	180.0
C3—C4—C5—C6	0.0	C2—C3—C8—O3	180.0
C3—C4—C5—C9	180.0	C4—C3—C8—O3	0.0
C2—C1—C6—C5	0.0	C6—C5—C9—O6	180.0
C7—C1—C6—C5	180.0	C4—C5—C9—O6	0.0
C4—C5—C6—C1	0.0	C6—C5—C9—O5	0.0
C9—C5—C6—C1	180.0	C4—C5—C9—O5	180.0

Symmetry codes: (i) *x*, −*y*+1/2, *z*.

### Hydrogen-bond geometry (Å, °)

**Table:**

*D*—H···*A*	*D*—H	H···*A*	*D*···*A*	*D*—H···*A*
O1—H1···O6^ii^	0.84 (3)	1.82 (3)	2.6435 (16)	165 (3)
O3—H3···O7^iii^	0.85 (4)	1.83 (4)	2.6593 (17)	164 (4)
O5—H5···O7^iv^	0.86 (3)	1.73 (3)	2.5723 (16)	169 (3)

Symmetry codes: (ii) *x*−1, *y*, *z*; (iii) *x*+1, *y*, *z*; (iv) *x*+1, *y*, *z*+1.
